# Adapting the Dietary Fat and Free Sugar Short Questionnaire: A Comprehensive Polish Modification for Enhanced Precision in Nutritional Assessments

**DOI:** 10.3390/nu16040503

**Published:** 2024-02-10

**Authors:** Katarzyna Tabiś, Monika Maćków, Dorian Nowacki, Ryszard Poprawa

**Affiliations:** 1 Institute of Psychology, University of Wroclaw, 50-137 Wroclaw, Poland; ryszard.poprawa@uwr.edu.pl; 2 Department of Human Nutrition, Wroclaw University of Environmental and Life Sciences, 50-375 Wroclaw, Poland; monika.mackow@upwr.edu.pl (M.M.); dorian.nowacki@upwr.edu.pl (D.N.)

**Keywords:** saturated fat, free sugar, adaptation, eating disorders, nutrition

## Abstract

Background: This study aimed to investigate whether The Dietary Fat and Free Sugar—Short Questionnaire (DFS) is a reliable and valid measure that can be used in Polish conditions. It involved 291 participants, aged 14 to 70 (M = 25.9, SD = 10.1), the majority of whom were women (75%). Methods: The questionnaire consisted of, among others, Polish DFS version, FFQ-6, TFEQ, and SCOFF. Test–retest reliability was established on the group of 26 students with a 2-week interval. Participants’ percentage of energy intake from both free sugars and saturated fats based on FFQ was correlated with DFS total and subscales scores. To investigate convergent and divergent validity, DFS scores with TFEQ correlations were performed. Diagnostic validity was established based on difference analysis between groups with the risk of eating disorders and those without the risk of those based on SCOFF. Results: Test–retest reliability (r_tr_ = 0.856) and internal consistency (α = 0.797) indicated excellent reliability. DFS correlated significantly with FFQ for both total scores (r = 0.82) and each subscale: sugar (0.79), fat (0.75), and fat–sugar (0.59). The correlations of DFS and TFEQ were statistically significant for cognitive restraint (r = 0.32) and uncontrolled eating (r = 0.19). There also have been found significant differences based on SCOFF regarding DFS. Conclusions: The results suggest good reliability and validity of the Polish version of DFS.

## 1. Introduction

The current pace of life, together with the costs of eating healthy, balanced meals, leads to the tendency to choose the fastest and most hearty meal. People would rather order the meal than do it themselves [1]. An increasing number of global deaths are caused by unhealthy diets [2,3], which is also crucial risk factors for noncommunicable diseases, including cancer, cardiovascular disease, type 2 diabetes, and obesity [4]. Recent systematic reviews suggest that obesity is highly linked to the consumption of sugar-sweetened beverages [5], as well as products containing saturated fats [6,7].

Regarding the influence of sugar on health, the implications of its harmfulness have already been considered in the conclusions of scientific research, including experimental research, in the 20th century [8,9]. Moreover, even the World Health Organization recommends reducing free sugar consumption to 10% of total energy intake or 5% to provide additional health benefits [4]. There is also growing evidence supporting the link between sugar consumption and alcohol addiction [10,11,12] and other mental health disorders [13,14]. Regarding saturated fats alone, there is still discussion regarding the legitimacy of saturated fat intake restriction [15,16]. Researchers suggest that we should not focus only on saturated fat itself but also on products that are a combination of saturated fat and free sugar, called palatable foods. These findings, among others, support the relevance of further investigation in the field of palatable food consumption, in terms of both somatic and mental health.

The evaluation of dietary habits is typically challenging because of the length and cognitive demands of common measures, such as the Food Frequency Questionnaire (FFQ), KomPAN, diet diaries, and 24 h recall. In Poland, one of the most commonly used measure of food intake is either FFQ or KomPAN. Both measures are highly demanding in terms of time and cognitive effort. While the FFQ is often used in cross-cultural comparison studies, KomPAN does not provide this option, as it is only available in Polish [17]. However, the availability of the Dietary Fats and Free Sugar—Short Questionnaire (DFS) provides researchers with a more concise option to select the most appropriate questionnaire for their study objectives, while also balancing the need for detail, accuracy, and broad population assessments. DFS is particularly valuable for researchers conducting studies on clinical samples, as the specific nature of this group does not allow for the use of long and time-consuming measures. Additionally, ethical considerations prohibit imposing significant effort on participants who are already experiencing suffering, as highlighted in the Helsinki Declaration [18]. Thus, the primary goal of this study was to develop and validate the Polish version of the DFS, which serves as a rapid and user-friendly screening tool with an easily adaptable scoring system for assessing the frequency of consuming products high in saturated fats and free sugars [19,20]. This innovative approach has the potential to enhance dietary interventions and inform public health policies, ultimately leading to improved outcomes.

The dietary practices and eating habits of individuals from Eastern Europe often involve overconsumption of saturated fatty acids, which are known to be significant risk factors for cardiovascular diseases [21,22]. These incorrect eating habits are prevalent in the region. Consequently, DFS can be utilized in other countries within our region that share similar dietary patterns and food choices.

## 2. Materials and Methods

### 2.1. Participants and Procedure

The current study is part of a research project that focuses on the meaning of sugar and fat consumption in mental health disorders, including alcohol dependence syndrome.

This study was conducted according to the standards laid down in the Declaration of Helsinki. The research project was carried out online from December 2021 to the end of January 2022 due to the SARS-CoV-2 pandemic (the lead author has full access to the data reported in this manuscript). The electronic version of the study was prepared using Google Forms and was available on researchers’ social media and online forums; however, it was also shared with students and transferred to other people.

First, translation and adaptation to Polish eating habits were conducted in collaboration with bilingual (Polish- and English-speaking) specialists of psychodietetics and clinical nutritionists. The process of adapting the contents of the items to the eating habits of the Polish population also involved integrating new (Polish-specific) products into the existing inventory and organizing them into existing subscales based on the primary nutrients they contained (mainly saturated fats, mainly free sugars or both). This categorization was based on the PZWL nutritional table. The final version of DFS can be found in the Supplementary Materials.

Next, the test–retest reliability was assessed in the sample consisting of N = 26 students (88.5% women) aged 18 to 35 years (*M* = 20.1) with a 2-week interval between the test and retest.

After evaluating the appropriate time stability, we commenced our study utilizing a larger participant pool of 291 individuals (69 males, 219 females, 3 not defined) aged 14 to 70 years (*M* = 25.9, SD= 10.1). The body mass index (BMI) varied from 15 to 40.6 (*M* = 22.5, SD = 4.05). BMI values were divided into six categories: underweight (0–18.49), normal weight (18.5–24.99), overweight (25–29.99), obesity I degree (30–34.99), obesity II degree (35–39.99), and extreme obesity (>40). Detailed sample characteristics can be found in Table 1.

### 2.2. Methods

The survey sheet comprised sociodemographic information, such as gender, age, education, and anthropometrics (measured by weight and height), as well as four separate questionnaires described below.

The Dietary Fat and Free Sugar—Short Questionnaire (DFS) is a 26-item self-report questionnaire designed to measure the consumption of products high in free sugar and saturated fat over the last 12 months [19]. The first 25 items concern the frequency of consuming products of different food groups. The last item concerns adding sugar to daily beverages or food. The answers were given on a scale ranging from 0 reflecting “*less than 1 per month*” to 4 reflecting “*more than 5 per week*”. For the last item, which relates to the number of teaspoons added to food and beverages, the scale ranges from 0, indicating “*none*,” to 4, indicating “*7+ teaspoons*”.

The items in DFS were divided into the following 3 groups (subscales): only saturated fats (F)—items 1–12; only free sugars (S)—items 17, 18, 20, 21, 23, 24, 26; saturated fat + free sugars (F + S)—items 13–16, 19, 22, 25. In the original study, DFS was scaled from 1 to 5, and the total score ranged from 26 to 130 points. In this study, each item was scored from 0 to 4. The total score of the Polish DFS can range from 0 to 104, where subscale results ranged: F from 0 to 48, S from 0 to 28, and F + S from 0 to 28.

The Food Frequency Questionnaire (FFQ) is a 62-item questionnaire including 62 food items and refers to the food consumption frequency over the last 12 months [23]. The answers were given on a scale ranging from “*never or very rarely*” (0 times/day), “*once a month or less*” (0.025 times/day), “*several times a month*” (0.1 times/day), “*several times a week*” (0.571 times/day), “*daily*” (1 time/day), or a “*few times a day*” (2 times/day). The Polish FFQ according to Wądołowska [23] showed good relative accuracy in estimating the daily food ration. FFQ responses were rated on a scale from 0 to 5 regarding the frequency of consumption, where 0 refers to “*never or almost never*” and 5 refers to “*several times a day*”. The items in the FFQ were also divided into the following groups: item group F—items 116, 24–28 52–57; item group S—items 5–8, 19, 39, 60–62; and item group F + S—items 9, 10, 13, 15. Taken together, the total score of the FFQ can range from 0 to 100, and the subscale results ranged from 0 to 48 for F, from 0 to 36 for S, and from 0 to 16 for F + S. Cronbach’s α for all FFQ-6 questions that measure the same variables as DFS in this sample was α_FFQ_ = 0.840.

The SCOFF Questionnaire (SCOFF) is a short, 5-item screening tool for eating disorders [24]. The core features concern anorexia nervosa and bulimia nervosa. Each question is rated with a yes or no answer, where “yes” is scaled as 1 and “no” as 0. The points from all items were summed, and a sum score greater than or equal to 2 indicates the likelihood of anorexia nervosa or bulimia.

The Three Factor Eating Questionnaire (TFEQ) is a short measure to assess eating behaviors, including cognitive restraint of eating, uncontrolled eating, and emotional eating [25]. It comprises 18 items, with the first 17 items rated on a 4-point Likert scale and the last item rated on an 8-point Likert scale. For analytical purposes, the answers to the first 17 items were recoded as follows: “*definitely yes”* = 3, “*rather yes”* = 2, “*probably not”* = 1, and “*definitely not”* = 0. Regarding the last item, the responses were categorized into four groups, with responses 1 and 2 recoded as 1, responses 3 and 4 as 2, responses 5 and 6 as 3, and responses 7 and 8 as 4.

### 2.3. Analysis

Statistical analyses were performed using STATISTICA 13 (TIBCO Software Inc., Palo Alto, CA, USA) and Jamovi (v. 2.3.18.0). For the reliability assessment, we used Cronbach’s alpha (α) as an internal consistency indicator and the test–retest correlation (r_tr_) as the time stability indicator. FFQ internal consistency was analyzed based only on questions that were categorized to reflect the same variables as DFS (F, S, F + S). It was calculated for both the DFS total score and FFQ total score (F, S, and F + S), and separately for each item group F, S, and F + S of DFS.

Both the convergent and divergent validity of DFS were assessed based on Spearman’s rank correlation with FFQ. The correlations of DFS with the FFQ between the score summaries and between the calculated kilocalories were analyzed.

Additionally, the validity was based on Spearman’s rank correlations between the frequency of consumption of saturated fats and free sugars (DFS) and eating behaviors such as cognitive restraint, uncontrolled eating, and emotional eating (TFEQ).

The diagnostic validity of DFS was assessed by comparing the frequency of saturated fat and free sugar consumption between individuals at risk of eating disorders, such as anorexia nervosa or bulimia nervosa, and those not at risk. For this purpose, the independent sample t-test and Mann–Whitney U test were used.

To estimate the dietary intake of free sugars and saturated fats in both questionnaires, we assigned a fixed number of servings to each answer regarding the frequency of consumption. In the FFQ, there were 0.25 servings posted to the answer “*never or almost never*”, to the answer “*once a month or less*”—0.625 servings, “*several times a month*” was 1.5 servings, “*few times a week*”—3.5 servings, “*daily*”—7 servings and “several times a day”—15 servings.

For DFS, “*less than once a month*” was assigned 0 servings, “*2–3 times a month*”—1.5 servings, “*1–2 times a week*”—5.5 servings, “*3–4 times a week*”—7 servings, and “*more than 5 times a week*”—14 servings. The last item of the DFS was the number of teaspoons added weekly to drinks, cereals, or food, for which we assigned 0 servings for “*none*” and 14 servings for “*7+*” teaspoons.

As mentioned above, both the DFS and FFQ were divided into three subscales: F, S, and F + S. With these scales, it was possible to calculate the energy value of a food ration from these three groups of products for Polish DFS, as presented in a previous adaptation research [19]. Using a fixed number of servings, the answers from the DFS and FFQ were converted into kilocalories (kcal) of saturated fatty acids and simple sugar daily consumption.

The total dietary intake from the FFQ was also estimated. The frequency (how often) and quantity (how much) of 43 food and beverage products consumed in the last four weeks were considered from the selected product groups. The percentage share of simple sugars and saturated fats from the FFQ-estimated daily food ratio was then calculated. Finally, the daily percentage of kilocalories of free sugars and saturated fats was calculated for the FFQ as well as for DFS using total dietary intake from the FFQ.

## 3. Results

The test–retest reliability ranged from 0.713 (F) to 0.786 (F + S), and for the total score r_tr_ = 0.856. Cronbach’s α for DFS, as a measure of internal consistency for the whole questionnaire without division into subscales, was 0.797. When divided into subscales, α for the fat subscale was α_F_ = 0.736, α_S_ = 0.594 for free sugar, and α_F+S_=0.637 for the sugar and fat subscale.

The normality assumption was violated for the DFS subscale scores based on the Shapiro–Wilk test (*p* < 0.05). However, the DFS total score distribution was similar to the normal distribution (*p* = 0.710). After testing for homogeneity of variances with Levene’s test, all variables, subscales, and total scores confirmed the assumption of equal variances (*p* > 0.05).

Intercorrelations of the DFS total score and each subscale were statistically significant, with r = 0.72 for the fat–sugar subscale, r = 0.76 for the sugar subscale, and r = 0.81 for the fat subscale, respectively. The correlations (regardless of sex) between DFS and FFQ were as follows: DFS total and FFQ total (r = 0.82, *p* < 0.001), DFS sugar and FFQ sugar (r = 0.79, *p* < 0.001), DFS fat and FFQ fat (r = 0.75, *p* < 0.001), and DFS fat–sugar and FFQ fat–sugar (r = 0.59, *p* < 0.001). Correlations (gender divided) of the total DFS score, the fat, sugar, and fat–sugar subscales with kilocalories of fatty acids and simple sugars daily consumption, daily percentage of kilocalories of saturated fats and free sugars, and target nutrient intake based on FFQ are presented in Table 2.

The total DFS score correlated significantly with sex (r = 0.20, *p* < 0.05) and age (r = −0.15, *p* < 0.05). Statistically significant differences across the genders were found regarding DFS total score and fat subscale. In the sugar subscale, there was a tendency toward statistical significance (see Table 3). Typically, men are more prone to consuming foods high in sugars and fats than women, with a particular emphasis on saturated fats.

In the entire sample (regardless of sex), BMI did not correlate significantly with the DFS total score (r = −0.02, *p* = 0.68), but it did correlate significantly (*p* < 0.05) with the DFS sugar subscale (r = −0.16), sex (r = 0.30), age (r = 0.37), and education (r = 0.26). BMI also correlated positively with all eating behaviors: cognitive restraint (r = 0.22, *p* < 0.001), uncontrolled eating (r = 0.20, *p* < 0.001), and emotional eating (r = 0.16, *p* = 0.008).

The group-wide correlations showed a statistically significant relationship between the DFS total score and cognitive restraint (r = −0.32, *p* < 0.001) and uncontrolled eating (r = 0.19, *p* = 0.002). DFS subscales correlated differently with eating behaviors. Cognitive restraint correlated significantly with all subscales: free sugar (r = −0.34, *p* < 0.001), saturated fat (r = −0.20, *p* < 0.001), and fat–sugar (r = −0.25, *p* < 0.001). Uncontrolled eating correlated significantly with the fat subscale (r = 0.13, *p* = 0.03) and fat–sugar subscale (r = 0.20, *p* < 0.001). Emotional eating did not correlate significantly with any of the DFS subscale scores.

The diagnostic result of SCOFF correlated significantly with the DFS total score (r = −0.18, *p* = 0.002), sugar subscale (r = −0.18, *p* = 0.003), and fat subscale (r = −0.24, *p* < 0.001). To confirm the diagnostic validity, an analysis of the differences between the group not showing the risk (*N* = 202) of eating disorders and the group with the risk of eating disorders (*N* = 86) was performed. The results indicated statistically significant differences between the groups in terms of saturated fat and total DFS score. Participants who were at risk of developing eating disorders tended to consume fewer items from the questionnaire than those who were not at risk. This finding was particularly notable for products that were primarily composed of saturated fats (F subscale) or free sugars (S subscale), as demonstrated in Table 3. The SCOFF diagnostic results correlated significantly with cognitive restraint (r = 0.20, *p* < 0.001), uncontrolled eating (r = 0.35, *p* < 0.001), and emotional eating (r = 0.35, *p* < 0.001).

## 4. Discussion

Our study aimed to evaluate the reliability and validity of the Dietary Fat and Free Sugar—Short Questionnaire in Polish conditions. After analyzing and adapting the content of the items of the questionnaire to Polish conditions, it was decided to split the item containing honey and peanut butter into two separate items to ensure greater transparency of our analysis. In the original paper, peanut butter and honey formed one item assigned to the fat–sugar subscale, but the dietitian collaborators in this study could not agree to place honey in this subscale because it does not contain saturated fats [19]. However, statistical analysis showed that the loading of the item containing nuts and peanut butter was negative, unlike the rest of the items, disturbing the internal consistency of the questionnaire. After removing this item from the questionnaire, the internal consistency increased significantly. This indicates the particularly important aspect of adapting a dietary tool to the customs of a given culture. Based on clinical observations, peanut butter is a product that does not appear too often in Polish tables; it is nutritionally niche and is consumed much less than in Anglo-Saxon countries or in other European countries. The report ‘EU—Peanut Butter and Prepared or Preserved Groundnuts—Market Analysis, Forecast, Size, Trends and Insights’, published in September 2020, showed that the countries with the highest consumption of peanut butter in 2019 were Great Britain (103 thousand tons), Germany (92,000 tons) and France (72,000 tons), with a combined share of 55% of total consumption. Poland, Spain, the Netherlands, Belgium, Portugal, Bulgaria, Sweden, Denmark, and the Czech Republic together accounted for 31% [26].

The reliability indicators, test–retest reliability, and internal consistency of the total DFS score, as well as the subscales, are acceptable and similar to the original and German versions of the tool [19,20]. Additionally, internal consistency was confirmed by strong and statistically significant intercorrelation of DFS total score with subscale scores.

As expected, men tend to eat generally more products listed in DFS. This result is in line with research that shows differences in food choices between genders [27]. According to the current study, men tend to choose products higher in fat, but they also eat sweet snacks more frequently. The WOBASZ II study, which included 5690 participants, indicated that men in Poland consume a higher proportion of energy from saturated fat in their diet than women [28]. Moreover, this finding suggests that the dietary habits of Poles diverge from those recommended for the prevention of cardiovascular diseases. Furthermore, several studies have examined the extent to which women regulate their body weight and pay attention to their food choices [29,30], which may explain why women generally consume fewer products listed on DFS compared to men. Our study produced a noteworthy finding: there was no statistically significant difference between genders in the consumption of foods that are high in both saturated fats and free sugars, which are commonly referred to as palatable foods. It was hypothesized that women, who typically pay more attention to their food choices, would consume less of these foods than men. However, upon examining the specific foods listed in the F + S subscale, it becomes evident that these products are often perceived as unhealthy and fattening (e.g., chocolate, cakes, pancakes, fast foods). Interestingly, the average score in the F + S subscale was less than 30% of the total points allocated to this subscale. This suggests that both men and women who participated in our study made an effort to reduce their consumption of the products listed in the F + S subscale, which may explain the lack of a significant difference in this subscale between genders.

The convergent validity of the tool was assessed based on DFS correlations with another measure that records the frequency and amount of food—FFQ [23]. Statistically significant and mainly strong correlations were found between the DFS total score and FFQ total score, as well as between corresponding item groups, which indicates that the questionnaire actually measured what was intended to be measured (see Table 2).

A correlation between the DFS total score and BMI was not found, which is congruent with reports from the English and German versions of the tool [19,20]. As mentioned in a previous publication regarding this tool, it might be expected that people with higher BMI would eat more products that are high in fats and sugars. However, research shows that obese people tend to choose low-energy-dense food instead of reducing the total amount of consumed food [31]. That tactic might not give them the expected outcomes, as we know from a meta-analysis of 8 trials, since the findings suggest that focusing solely on reducing sugar-sweetened beverage (SSB) intake does not show consistent correlations with changes in BMI [32]. This might also be the reason for the negative relationship between BMI and the DFS sugar subscale, as it was the only statistically significant correlation of BMI with DFS. The theory suggests that people with higher weight tend to differ from those with “normal” weight in being constantly on a diet [33]. This might lead them to unconstructive eating behaviors.

The results of this study also confirm this theory, as BMI correlates positively with all measured eating behaviors, including cognitive restraint, uncontrolled eating, and emotional eating. As the research shows, restrained eating might lead to the loss of control due to disinhibited consumption of too many products [34] and higher BMI [35,36]. This is explained by the framework of “restraint theory” [33,34], which assumes that cognitive restraint is under cognitive control, which means eating to meet dietary requirements that do not respond to physiological cues. Because it disbalances the perception of satiety and hunger, restrained eating might lead to cognitive abandonment of the dietary rules and result in the increased consumption of less healthy products [37]. This cause-and-effect theoretical sequence easily explains the positive correlation of DFS total score with uncontrolled eating, which might simply be the repercussion of lost cognitive control for cognitively restrained individuals.

The DFS validity was also confirmed by the correlations of DFS with the TFEQ. The results suggest that the DFS total score correlates moderately negatively with cognitive restraint and weakly positively with uncontrolled eating. It is known that cognitive restraint is associated with a greater risk of binge eating or eating when experiencing negative emotions [33]. People using cognitive restraint invest a great amount of energy to fight the urge to eat or achieve their desired figure. Cognitive restraint, as previously mentioned, is an eating behavior under cognitive control [33,34] that incorporates dietary restrictions. Dietary restrictions might be applied to all products listed in the DFS, but even WHO requirements [38] suggest reducing sugar consumption, and it is well known that foods high in both fats and sugar together are considered unhealthy and increase hunger for more [39]. This might also be the reason for the positive correlation between uncontrolled eating and the fat–sugar subscale.

The last part of the current study concerned analyzing diagnostic validity based on the difference between individuals at risk of eating disorders and those not at risk, in terms of DFS total score and subscales. The differences between groups were statistically significant regarding DFS total, fat subscale, and sugar subscale. All these results indicated that individuals at risk of anorexia nervosa or bulimia nervosa consumed products listed in the whole questionnaire, as well as those containing mainly saturated fats or free sugars, less frequently. There is also a statistically significant correlation between SCOFF and uncontrolled, emotional eating. This might be the basis for understanding why there is no significant correlation with the fat–sugar subscale. The evidence shows that a diet high in saturated fats and sugars is associated with changes in central dopamine levels [40]. Eating products high in both saturated fat and free sugar might be understood as comfort eating—a form of emotion regulation, which is important in understanding what underlies eating disorders [41]. However, the current study sample did not involve a clinical sample, so although the group at risk of eating disorder nominally consumed more high-fat–sugar products than the group without the risk, the difference between the groups might not be strong enough to be statistically significant.

Future studies on the Polish version of DFS should also investigate the discrepancies in dietary habits among individuals from various regions of Poland. Assessing the variations in the consumption patterns of foods rich in saturated fats and free sugars could offer valuable insights into the validity of DFS and aid in the development of targeted preventive measures and nutritional interventions to mitigate the risk of noncommunicable diseases in the respective regions.

### Study Limitations

Our investigation was carried out on a group of voluntary participants with a median age of approximately 22 years. It would be beneficial to conduct further research using DFS on a more age-diverse sample. The DFS tool is subject to the limitations inherent in self-report measures, which constitute a significant drawback when interpreting the outcomes. Consequently, it is essential to bear these shortcomings in mind when examining the results. 

## 5. Conclusions

The results obtained in the current study indicate that the Polish version of DFS is a psychometrically correct measure to assess the frequency of consuming products high in saturated fats and free sugars. The total score of DFS might be a better indicator for the consumption of those products than each subscale separately. It is a quick and easy-to-administer self-report questionnaire that might be especially useful for clinicians and researchers in Poland.

## Figures and Tables

**Table 1:** Participants’ Characteristics (*N* = 291).

Education			Gender			BMI Category		
	*n*	%		*n*	%		*n*	%
Basic	3	1	Woman	219	75.3	0–18.49	30	10.3
Vocational	1	0.3	Men	69	23.7	18.5–24.99	198	68
Secondary	186	63.9	Not defined	3	1	25–29.99	46	15.8
Higher	101	34.7				30–34.99	11	3.8
						35–39.99	5	1.7
						40+	1	0.3

**Table 2:** The Spearman’s rank correlations for DFS score and subscales with nutrient estimates and scores from the FFQ.

	Gender	Men	Woman	Men	Woman	Men	Woman	Men	Woman
Variables		DFS_S_		DFS_F_		DFS_F + S_		DFS_total_	
FFQ S		0.76 *	0.79 *	0.34 *	0.41 *	0.48 *	0.56 *	0.72 *	0.72 *
FFQ F		0.36 *	0.31 *	0.66 *	0.74 *	0.37 *	0.32 *	0.69 *	0.63 *
FFQ F + S		0.22 ^ns^	0.47 *	0.20 ^ns^	0.40 *	0.48 *	0.61 *	0.39 *	0.62 *
FFQ total		0.61 *	0.60 *	0.56 *	0.72 *	0.51 *	0.54 *	0.80 *	0.81 *
FFQ SF (kcal)		0.12 ^ns^	0.28 *	0.57 *	0.62 *	0.33 *	0.36 *	0.51 *	0.56 *
FFQ FS (kcal)		0.31 *	0.24 *	0.33 *	0.16 *	0.32 *	0.31 *	0.45 *	0.28 *
FFQ FS %		0.18 ^ns^	−0.08 ^ns^	−0.27 *	−0.42 *	0.09 ^ns^	−0.05 ^ns^	−0.04 ^ns^	−0.28 *
FFQ SF %		−0.23 ^ns^	0.20 *	0.30 *	0.48 *	0.14 ^ns^	0.23 *	0.12 ^ns^	0.42 *

Note: DFS_S_—sugar subscale of DFS; DFS_F_—fat subscale of DFS; DFS_F + S_—fat and sugar subscale of DFS; DFS_total_—total score of DFS; FFQ S—items consisting mainly sugar in FFQ; FFQ F—items consisting mainly fat in FFQ; FFQ S + F—items consisting mainly sugar and fat in FFQ; FFQ total—total score for items of FFQ; FFQ SF (kcal)—kcal from saturated fats in FFQ; FFQ FS (kcal)—kcal from free sugars in FFQ; FFQ FS %—daily percentage of kilocalories of free sugars in FFQ; FFQ SF %—daily percentage of kilocalories of saturated fats in FFQ; * *p* < 0.05, ^ns^ not significant.

**Table 3:** Difference analysis across gender and risk of eating disorders with group descriptives.

	Grouping Variable	Mean	K-S	Statistic	Grouping Variable	Mean	K-S	Statistic
DFS_S_	Men	8.46	*p* > 0.10	−1.79 ^t^	Risk	6.66	*p* < 0.025	2.92 **
	Woman	7.43			No risk	8.11		
DFS_F_	Men	16.57	*p* < 0.05	−3.98 ***	Risk	12.16	*p* < 0.001	4.05 ***
	Woman	13.41			No risk	15.02		
DFS_F+S_	Men	7.38	*p* > 0.10	−1.08 ^ns^	Risk	7.19	*p* > 0.1	−0.67 ^ns^
	Woman	6.84			No risk	6.88		
DFS_total_	Men	32.41	*p* < 0.05	−3.45 ***	Risk	26.01	*p* < 0.025	3.08 **
	Woman	27.68			No risk	30		

Note: ** *p* < 0.01, *** *p* < 0.001, ^t^ *p* < 0.1, ^ns^ not significant; statistic—Student’s *t*-test value if Kolmogorov‒Smirnov test is not statistically significant and Mann–Whitney U test value if Kolmogorov‒Smirnov test is statistically significant.

## Data Availability

The data presented in this study are available on request from the corresponding author.

## Supplementary Materials

The following supporting information can be downloaded at: https://www.mdpi.com/article/10.3390/nu16040503/s1. The Dietary Fat and free Sugar—Short Questionnaire.
