# Supplementary material for: Adapting the Dietary Fat and Free Sugar Short Questionnaire: A Comprehensive Polish Modification for Enhanced Precision in Nutritional Assessments
Source: Nutrients. 2024 Feb 10;16(4):503. doi: 10.3390/nu16040503 (PMC10892036; doi:10.3390/nu16040503)
Supplement: Supplementary file 1 [file nutrients-16-00503-s001.zip › nutrients-2825250-supplementary.pdf]

Zawartość cukrów i tłuszczy w diecie – krótki kwestionariusz*

The Dietary Fat and free Sugar – Short Questionnaire

H. Francis i R. Stevenson

Pomyśl o produktach, które spożywałeś przez ostatni rok. Uwzględnij wszystkie posiłki: śniadania, drugie śniadanie, lunch, obiady, podwieczorki, kolacje. Przy każdym z poniżej wymienionych produktów i napojów oceń, jak często go spożywałeś, wskazując tylko jedną odpowiedź.

		rzadziej niż 1 raz w miesiącu	2-3 razy w miesiącu	1-2 razy w tygodniu	3-4 razy w tygodniu	częściej niż 5 razy w tygodniu
F	1.Mielone mięso wołowe lub jagnięce, np. jako kotlet, sznycel, w hamburgerach czy spaghetti bolognese					
F	2. Wołowina lub wieprzowina jako stek, żeberka czy pieczeń					
F	3. Mięso z kurczaka w każdej postaci					
F	4. Kiełbasy, kabanosy, parówki, frankfurterki lub salami					
F	5. Boczek (bekon)					
F	6. Sosy sałatkowe					
F	7. Margaryna, masło, smalec lub olej do smażenia					
F	8. Jajka (np. gotowane, smażone)					
F	9. Pizza					
F	10. Ser żółty (również topiony i smażony), pleśniowy, twaróg półtłusty i pełnotłusty					
F	11. Placki ziemniaczane, smażone ziemniaki lub frytki					
F	12. Chrupki, chipsy, popcorn, paluszki czy nachosy					
F+S	13. Pączki, ciastka, rogaliki, bułki słodkie					
F+S	14. Torty, ciasta					
F+S	15. Lody (oprócz sorbetów lub lodów niskotłuszczowych)					
F+S	16. Czekolada i wyroby czekoladowe (krem czekoladowy, batony, czekoladki)					
S	17. Lizaki, cukierki, żelki					
S	18. Słodkie smarowidła, np. dżem, miód, powidła					
F+S	19. Naleśniki, racuchy, panckes, tosty francuskie, omlet					
S	20. Napoje energetyzujące (np. RedBull, Monster, Tiger)					
S	21. Słodkie napoje gazowane (oprócz napojów typu *light* czy zero)					
F+S	22. Pełnotłuste mleko (oprócz napojów roślinnych) lub jako dodatek do kawy, herbaty, shaków czy kakao					
S	23. Inne słodkie napoje np. soki, nektary, kompot lub herbata czy kawa z dodatkiem cukru					
S	24. Białe pieczywo (np. chleb, bułki, bagietki)					
F+S	25. Jak często zamawiałeś jedzenie w lokalach szybkiej obsługi (*fast food*) w ciągu ostatniego roku?					
		Żadnej	1-2	3-4	5-6	7+
S	26. Ile łyżeczek cukru dodałeś w ciągu ostatniego tygodnia do swojego jedzenia, napojów, płatków śniadaniowych?					

* Polska adaptacja, S- free sugar subscale (wolne cukry), F- saturated fats subscale (tłuszcze nasycone), F+S – fat and sugar subscale (cukry i tłuszcze)

The Dietary Fat and Free Sugar – Short Questionnaire

English translation of Polish adaptation[1]

Think about the foods you have consumed over the past year. Include all meals: breakfast, brunch, lunch, dinner, afternoon tea, and supper. For each of the following foods and drinks, evaluate how often you consumed it, indicating only one answer.

		Less than 1 per month	2-3 per month	1-2 per week	2-3 per week	5+ per week
F	1.Minced beef or lamb, for example as a cutlet, schnitzel, in hamburgers or spaghetti bolognese					
F	2.Beef or pork as steak, ribs, or roast					
F	3.Chicken meat in any form					
F	4.Sausages, "*kabanosy*", weenie, frankfurters or salami					
F	5.Bacon					
F	6.Salad dressings					
F	7.Margarine, butter, lard or frying oil					
F	8.Eggs (e.g., boiled, fried)					
F	9.Pizza					
F	10.Cheese (also spread and fried), blue cheese, semi-skimmed and full farmer's cheese					
F	11.Potato cakes, fried potatoes or fries					
F	12.Crisps, corn crunchies, popcorn, salty sticks or nachos					
F+S	13.Donuts, biscuits, croissants, sweet rolls					
F+S	14.Cakes, pies, pastries					
F+S	15.Ice cream (except sorbets or skimmed ice cream)					
F+S	16.Chocolate and chocolate goodies (chocolate cream, bars, chocolates)					
S	17. Lollipops, candies, jelly candies					
S	18.Sweet spreads, such as jam, honey, plum jam					
F+S	19.Crepes, apple crumpets, pancakes, French toast, omelet					
S	20.Energy drinks (e.g. RedBull, Monster, Tiger)					
S	21.Sodas (except light soda)					
F+S	22.Full milk (except non-dairy milk) or as an addition to coffee, tea, shakes or hot chocolate					
S	23.Other sweet drinks such as juices, nectars, „*kompot*" or tea or coffee with added sugar					
S	24.White breadstuff (e.g. bread, bun, baguettes)					
F+S	25.How often have you ordered fast food in the last year?					
		None	1-2	3-4	5-6	7+
S	26.How many teaspoons of sugar have you added in the last week to your food, drinks, breakfast cereals?					

[1] This is only a translation of Polish DFS content. It cannot be used in research in this English version. For the validated English version, please use Francis and Stevenson (2012).
